# Synthesis and characterisation of microporous, conducting, photoactive novel pyrene containing material

**DOI:** 10.1039/d6ra02730j

**Published:** 2026-04-07

**Authors:** Adam Rowling, Julien Doulcet, Ellena Sherrett, Robert Dawson, Michael J. G. Peach, Sam Harley, Benjamin Robinson, Abbie Trewin

**Affiliations:** a Department of Chemistry Lancaster University Bailrigg Lancaster LA1 4YB UK a.trewin@lancaster.ac.uk; b Department of Chemistry Dainton Building, 13 Brook Hill Sheffield S3 7HF UK; c Department of Physics, Lancaster University Bailrigg Lancaster LA1 4YB UK

## Abstract

Here we report a novel framework material, OSPC-Py, synthesised *via* an organic one-pot route, highlighting its structural modularity and potential for further chemical functionality to be incorporated. It is highly microporous with a SA_BET_ = 850 m^2^ g^−1^, is a semi-conductor with a conductivity of 1.32 × 10^−9^ S cm^−1^, and is photo-active. Demonstrating the novel potential to incorporate optically active chemical functionality within an semi-conductive yet microporous material. This is a very attractive target with many potential applications, including photochromism, photomodulated luminescence, and photocatalysis.

## Abstract 2

OSPC-Py – a novel framework material that is microporous, a semi-conductor, and photoactive, synthesised *via* an organic one-pot route highlighting the structural modularity and potential for further functionality.

## Graphical abstract

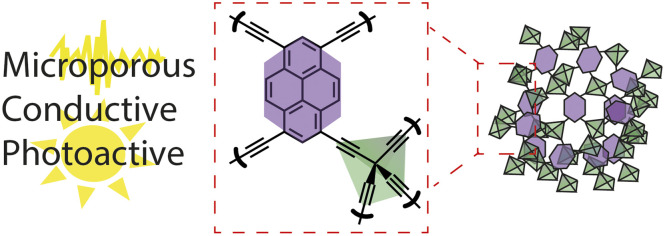

## Introduction

Conjugated microporous polymers (CMPs)^1,2^ are amorphous frameworks where inefficient packing creates pores throughout the material. They have extended π-conjugation that enables CMPs to exhibit interesting electronic properties and a broad range of applications. For example, the optical band gap was fine tuned in a library of CMP materials through precise statistical copolymerisation,^3^ and the molecular detection sensitivity of a 3,8,13-tribromo-5,10,15-triethyltriindole derived CMP was increased in comparison to a linear polymer analogue.^4^

Pyrene is an attractive chemical functional group to incorporate into polymeric materials due its optical properties and potential for further tuning through chemical functionalisation. Pyrene-based CMPs are relatively chemically simple highly-conjugated photo-active polymers (an ability to interact with light), first introduced in 2011 by Jiang *et al.*^5^ In particular, a series of pyrene-based CMPs were produced *via* the use of 1,3,6,8-tetrabromopyrene as a monomer.^3,5–8^

While porous and electronically conductive organometallic materials are known, including metal organic frameworks (MOFs),^9^ hybrid materials,^10^ and carefully synthesised covalent organic framework films,^11^ amorphous microporous polymer materials with these properties are less common. Therefore, a fully organic, photo-active electronically conductive, and porous material is an attractive target with many potential applications, including photochromism, photomodulated luminescence, and photocatalysis.^12^

Organically synthesised porous carbons, or OSPCs, are a new and exciting class of porous materials within the CMP family that combine high surface areas with electrical conductivity and chemical and thermal robustness.^13–15^ The first OSPC, OSPC-1, attracted significant interest for its performance as a Li-Ion battery anode and its status as a novel allotrope of carbon. However, the complicated multi step synthesis and low stability of reactants led to its limited potential for further exploration. An alternative one-pot synthesis method was reported for OSPC-1 (and a new family member, OSPC-0), that has the same polymeric structure and electrochemistry as when synthesised by the original multi-step method.^16^ This opened a pathway towards a new family of functionalised OSPC like materials.

The alternative route used in the one-pot process whereby the ethynyl struts are added to a nodal carbon allows for additional functionalities to be introduced into an OSPC framework, providing they are capped with two or more silyl protected ethynyl groups.

**Fig. 1:**
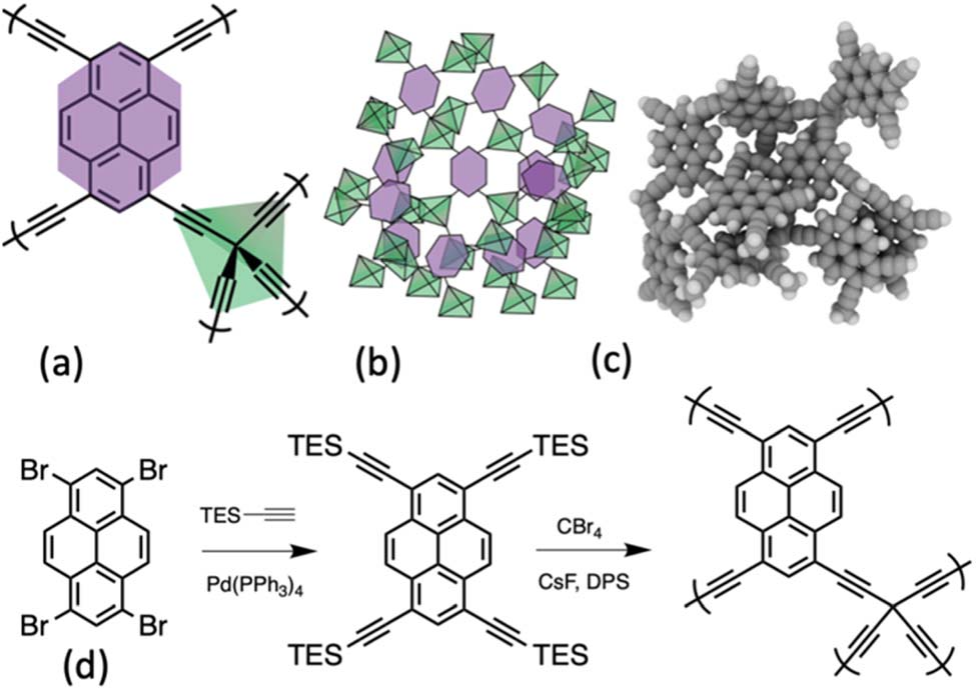
(a) The polymeric structure of OSPC-Py, (b) a cartoon of the 3-D structure, and (c) a cluster model with 20 pyrene or OSPC building blocks visualised using iRASPA.^17^ (d) Synthesis of OSPC-Py (see SI section 3 for details).

Therefore, combining the properties of pyrene with the electronically conductive OSPC material is a very appealing objective. Herein we describe the synthesis and characterisation of a novel framework material, OSPC-Py (Please see SI section 3.2 for a discussion on naming rationale). Whereby a tetraethynylpyrene co-monomer has four ethynylstruts that are available to react with the tetrabromomethane co-monomer resulting in a hyper cross-linked polymer structure with tetrahedral sp^3^ carbon nodes linked *via* an ethynyl strupyrene–ethynyl strut, shown in Fig. 1.

## Results and discussion

OSPC-Py was synthesized through the one pot method outlined in SI section 3.^18^ Solid state NMR (SSNMR) (Fig. SI.1) showed a very similar spectra to that observed for OSPC-1 and OSPC-0.^18^ A peak is observed at 128 ppm visible above an underlying broad peak from 112 – 152 ppm. Previous work has identified the broad peak in this region as being due to interacting sp C from closely packed ethynyl chains within the OSPC material and the sharper peak at 128 ppm as being due to pyrene unit within a CMP network.^18^ We therefore assign the peaks in this region as being due to the pyrene unit and ethynyl C within the OSPC-Py. Similarly, a peak at 55 ppm was identified as being due to the sp^3^ C in OSPC networks and so we identify the peak at 50 ppm as being due to the sp^3^ C within the OSPC-Py.^18^

UV-vis data collected, shown in Fig. SI.2, shows a very broad absorption ranging from ∼200 nm to 800 nm (the full range recorded). This is in-line with the black colour of the material and common to highly conjugated carbon-based materials.^19^

**Fig. 2:**
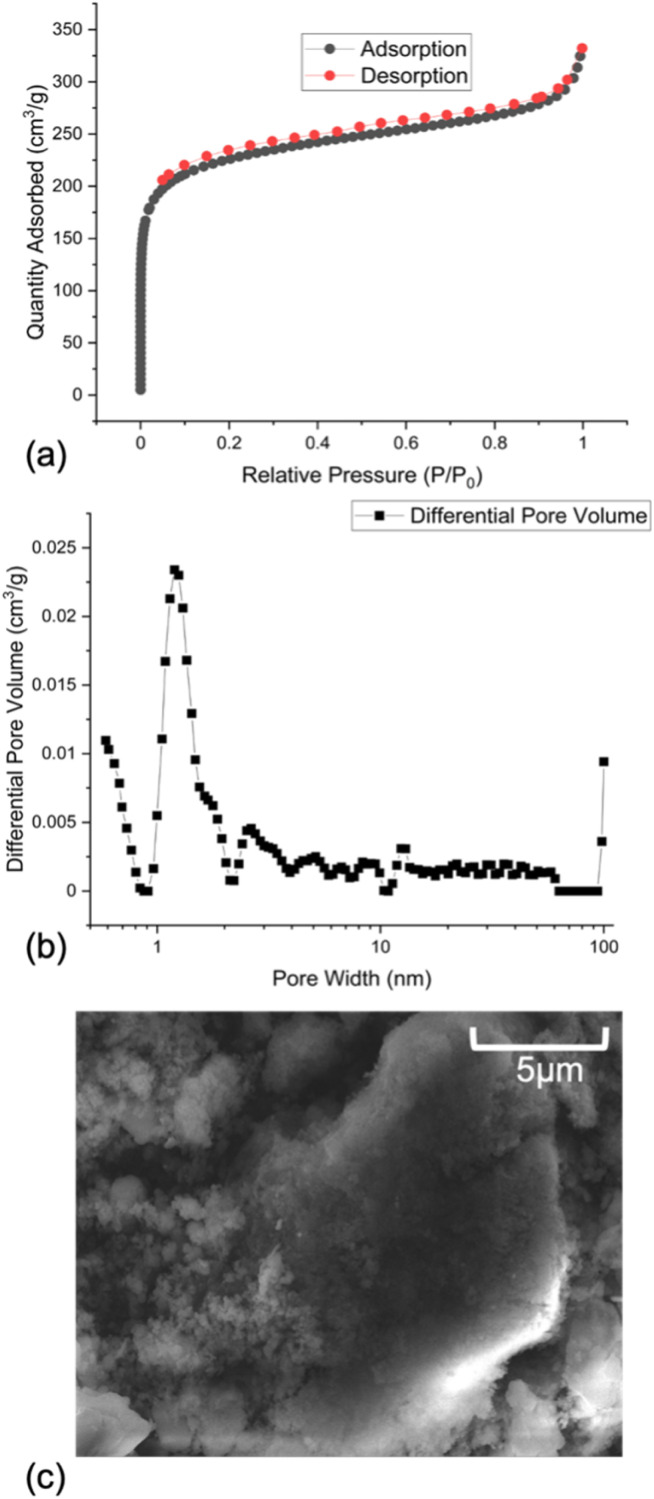
(a) Nitrogen gas adsorption isotherm, (b) pore size distribution, and (c) SEM.

Surface area and porosity analysis was performed on OSPC-Py through N_2_ sorption (Fig. 2(a)). BET surface area analysis was undertaken through a full isotherm analysis giving a relatively high surface area of 850 m^2^ g^−1^. The isotherm recorded is type II/IV in appearance. The pore size distribution shows similarities to previously reported OSPC materials with a large peak at ∼1.2 nm which correlates to the C sp^3^-to-C sp^3^ distance within OSPC-Py.^16^ A clear shoulder to this peak is observed at 1.75 nm, which correlates to pyrene-to-pyrene distances observed in cluster models, as shown in Fig. SI.3.

High resolution SEM imaging, shown in Fig. 2(b) and SI.4, show two morphologies present, a dense smooth morphology and a fuzzy fluffy region. EDX, Fig. SI.5, confirms that chemical composition in both regions is identical and corresponds to the expected atomic weight percentages for OSPC-Py. These two distinct but chemically identical morphologies have been observed for other OSPC materials and described as being due to dense regions where the ethynyl groups are in close contact and so are highly interacting, and open framework regions.^16^ The dense region was identified as being dominant on the solid state NMR due to CSA effects in the open framework regions and so not being visible in the solid state NMR. Whereas the open framework regions are highly porous and are responsible for the high porosity observed. We therefore believe that the same interpretation can be made for OSPC-Py and rationalises the SSNMR and porosity data.

**Fig. 3:**
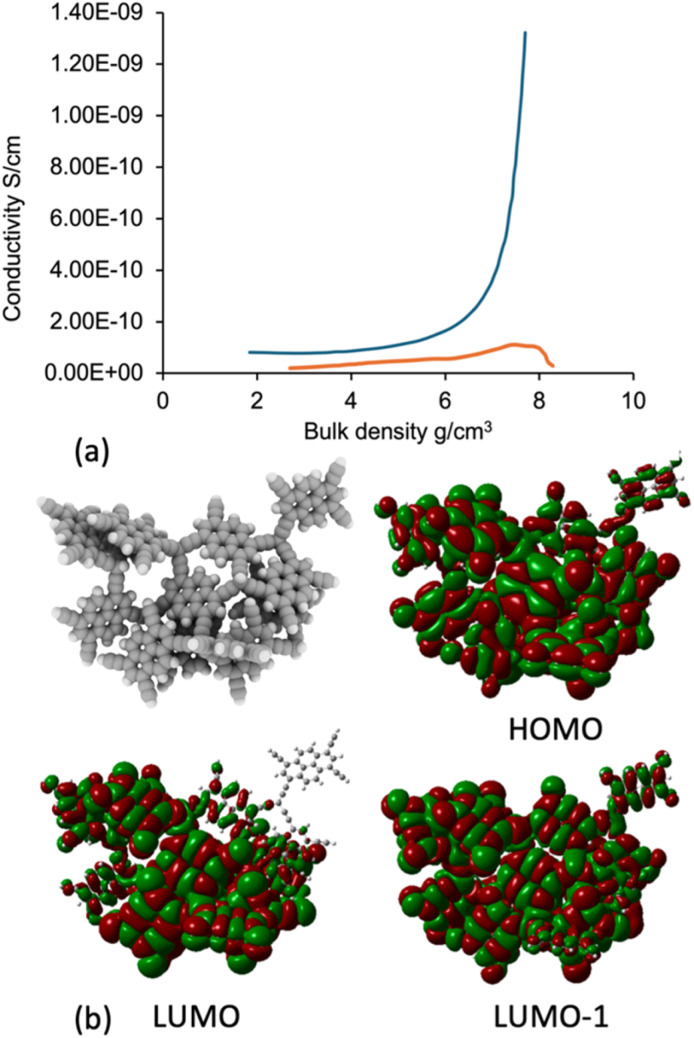
(a) Conductivity of OSPC-Py (blue) compared to a non-conductive sample of cellulose (orange) (b) molecular orbital of a Webbed cluster model.

The electrical conductivity was measured as a function of pressure, shown in Fig. 3(a), from 0 to 700 MPa using a hydraulic press (SA9003, Hioki E.E. Corporation) with an impedance analyser (IM3570, Hioki E.E. Corporation). OSPC-1 is known to be electrically conductive with a conductivity of 1.2 × 10^−4^ S cm^−1^, determined from a Nyquist plot.^13^ This approach uses a frequency-swept voltage source and so results in high conductivities due to the inclusion of other properties, including capacitance, at higher frequencies. Here, we use a fixed low-frequency (4 Hz) voltage source to more directly measure the electrical conductivity of the material without inclusion of these other influences. This means that a direct comparison between materials can be undertaken. Using this approach, OSPC-Py demonstrated a peak conductivity of 1.32 × 10^−9^ S cm^−1^. This is comparable to other semi conductive organic materials, although below values seen for other carbon-based material such as polyacetylenes and polythiophene.^20–22^ A clear difference in behaviour is observed between OSPC-Py and a non-conductive cellulose sample used as a comparator.

Previously, the electron transport mechanism in OSPC-1 was determined to be *via* phase-coherent tunnelling confirmed by wavefunction analysis that showed clear conduction pathways along the OSPC-1 framework, particularly in the HOMO(H1) and LUMO (L0 and L1) levels. To assess the potential for these pathways to exist within OSPC-Py, we generated 100 20-unit cluster models using our in-house GPU-based Ambuild code, written in Python and specifically designed to model amorphous porous polymers. For geometry optimization and molecular dynamics (MD), Ambuild integrates with HOOMD-blue and uses the Polymer Consistent Forcefield (PCFF).^23–26^ Once generated, The GFN2-xTB^27^ semi-empirical tight binding approach was used within the XTB^28^ program to optimize the geometry of the Ambuild-generated clusters. Finally, a single point energy calculation was undertaken for each model using Gaussian 09 with the CAM-B3LYP/def2SVP model chemistry to generate molecular orbitals.^29–31^ See SI Section 4 for full computational details.

The set of 100 cluster models were assessed for the number of macrocyclic rings (MCRs) finding that most had at least 1 MCR and a high proportion having 2, 3, and 4 MCRs. The maximum number of MCRs found was 7. 82 of the 100 models showed π–π stacking between pyrene units. 4 broad classifications were identified being branched (no MCRs), Single (1 MCR), webbed (pyrene units planar orientation relative to each other joined through sp^3^ C), and disjointed (pyrene units are perpendicular to a webbed region). The Webbed classification was dominant with 81 members. Examples are shown in SI Section 8.

Similarly to OSPC-1, we see clear overlap of H1, L0, and L1 molecular orbitals for the OSPC-Py cluster models, shown in SI section 8. Fig. 3(b) highlights this for a Webbed model. Molecular orbitals are seen to extend across multiple pyrene and sp^3^ C units in the HOMO, LUMO, and LUMO(1), providing a clear potential pathway for electron transport.

## Conclusions

In conclusion, we have synthesised a new material that incorporates a photoactive pyrene unit, is highly porous, and is electrically conductive. We have rationalised the electronic conductance through analysis of cluster models showing clear conductance pathways through molecular orbitals that traverse multiple pyrene and sp^3^ C units. This opens up a new and exciting direction through further chemical functionalisation to specify and tailor band gaps to novel materials for specific applications.

## Author contributions

Adam Rowling and Julien Doulcet: synthesis, data collection and analysis, Michael J. G. Peach and Ellena Sherrett: computational data collection and analysis, Robert Dawson: porosity data collection and analysis, Sam Harley and Benjamin Robinson: conduction data collection and analysis, Abbie Trewin: conceptualisation, editing, data analysis, data collection and supervision. All authors contributed to manuscript drafting.

## Conflicts of interest

There are no conflicts to declare.

## Supplementary Material

RA-016-D6RA02730J-s001

## Data Availability

The data supporting this article have been included as part of the supplementary information (SI). The code for Ambuild can be found at https://github.com/linucks/ambuild. Supplementary information is available. See DOI: https://doi.org/10.1039/d6ra02730j.
