# Supplementary material for: Synthesis and characterisation of microporous, conducting, photoactive novel pyrene containing material
Source: RSC Adv. 2026 Apr 7;16(20):18283–6. doi: 10.1039/d6ra02730j (PMC13055586; doi:10.1039/d6ra02730j)
Supplement: RA-016-D6RA02730J-s001 [file RA-016-D6RA02730J-s001.pdf]

Supplementary Information (SI) for RSC Advances.
This journal is © The Royal Society of Chemistry 2026

Synthesis and characterisation of microporous, conducting, photoactive novel pyrene based OSPC

Adam Rowling,[a] Julien Doulcet,[a] Ellena Sherrett,[a] Robert Dawson,[b] Michael J. G. Peach,[a] Sam Harley,[c] Benjamin Robinson,[c] and Abbie Trewin[a*]

Supporting Information

Contents

1. **Instruments**
    1. **Solid state NMR**
    2. **Gas sorption**
    3. **SEM and EDX**
    4. **UV/vis**
    5. **Conductivity**
    6. **CHNS analysis**
    7. **TGA**
2. **Materials**
3. **Synthetic procedures**
    1. **Synthesis of 1,3,6,8-Tetra(triethylsilylethynyl) pyrene**
    2. **Synthesis of OSPC-Py**
4. **Computational generation of OSPC-Py cluster models**
5. **Solid state NMR of OSPC-Py**
6. **UV/vis**
7. **SEM images**
8. **EDX**
9. **Cluster models**

1. Instruments

1.1 Solid state NMR

Solid state NMR (SSNMR) was performed on a Bruker AVANCE III HD 700Wb. The results were obtained through cross polarisation of ^{13}C with ^{1}H. The MAS frequency was 16 kHz and the D1 was 3s.

1.2 Gas sorption

Polymer surface areas and pore size distributions were measured by nitrogen adsorption and desorption at 77.4 K using a Micrometrics 3-Flex adsorption analyser over a relative pressure range (P / P_0) of 0.01 to 0.25. Before the analysis, the materials were outgassed under a primary vacuum at 100 °C for 24 hours.

1.3 SEM and EDX

SEM was performed on a Field-free UHR-SEM BrightBeam of the TESCAN AMBER model. Electron backscatter diffraction was performed with an Oxford Instruments Symmetry S3. EDS was performed on an Oxford Instruments Ultim Max 40 using a 40 mm^2 large area EDS detector. Images were captured at between 100× and 30000× magnification at 2.00 kV acceleration. Powders were dropped onto carbon tabs ((G3348N, Agar Scientific) for this measurement.

1.4 UV–vis

All absorption spectra were recorded in a UV–vis absorption spectrometer (Cary 60, Agilent) in the spectral range 200−800 nm using a solid sample holder. Reference spectra were obtained in an identical sample holder with a PTFE 200-800 reference sample.

1.5 Conductivity

The electrical conductivity was measured as a function of pressure from 0 to 700 MPa using a powder impedance measurement system (Hioki E.E Corporation). Conductivity was

measured in a 2-probe configuration using an AC impedance analyser (IM3570, Hioki E.E Corporation), with a fixed bias modulation frequency of 4 Hz and amplitude of 0.5 V.

1.6 CHNS analysis

CHNS analysis was performed on a Vario MICRO cube using 2 mg samples.

1.7 TGA

Thermogravimetric analysis was performed on a NETZSCH STA 449F3 thermal analyser using alumina crucibles. The sample mass was 12.67 mg, and it was heated from 50 °C to 900 °C at a rate of 10 °C/min

2. Materials

1,3,6,8-Tetrabromopyrene was obtained from TCI, Copper Iodide, Cesium Floride, and diphenylsulfone were obtained from Alfa Aesar. Tetrakis(triphenylphosphine) palladium(0), triethylamine, and triethylsilyl acetylene were obtained from Fluorochem. Carbon tetrabromide was obtained from Sigma Aldrich. All solvents (including anhydrous toluene Acroseal) were obtained from Fisher Scientific.

Thin layer chromatography was performed on aluminium sheets coated with Merck silica gel 60 F254 with visualisation using potassium permanganate solution, phosphomolybdic acid, and/or scrutinised under 254 nm UV light. Column chromatography was performed using Silica 60 (40–63 microns) supplied by Sigma-Aldrich or Fluorochem unless otherwise stated. Microwave vials (crimp cap seal) were purchased from Biotage.

Nuclear magnetic resonance (NMR) spectroscopy was performed on a Bruker Avance 400 NMR spectrometer (^{1}H NMR at 400 MHz, ^{13}C NMR at 100 MHz) with the appropriate deuterated solvent. Chemical shifts in ^{1}H NMR and ^{13}C NMR spectra are relative to the deuterated solvent peak and reported as singlet (s), doublet (d), triplet (t), quartet (q) and combinations thereof, or multiplet (m). Coupling constants (*J*) are quoted in Hz and are averaged between coupling partners and rounded to the nearest 0.1 Hz. FT-IR data was acquired using Agilent Technologies Cary 630 FTIR instrument with wavenumbers being reported in cm^{-1}. Melting points were determined using a Gallenkamp melting point apparatus with in with a mercury thermometer.

3. Synthetic procedures

3.1 Synthesis of 1,3,6,8-Tetra(triethylsilylethynyl) pyrene

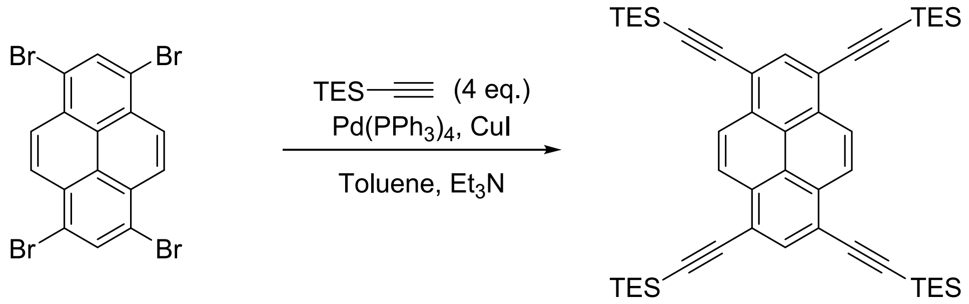

According to the method of Lotsch and co-workers.[1a] To a solution of 1,3,6,8-tetrabromopyrene (518 mg, 1 mmol 1 e.q.) in dry toluene (4 mL) and dry triethylamine (12 mL, 9 mmol) was added $Pd(PPh)_4$ (173 mg, 0.15 mmol 0.15 e.q.) and CuI (60 mg 0.3 mmol 0.3 e.q.) under anhydrous conditions. The resulting mixture was heated to 50 ˚C, where TES acetylene (1 mL 9 mmol 9 e.q.) was added dropwise, and then to 80 ˚C where it was left under reflux overnight. The reaction mixture was diluted with dichloromethane (48 mL) and filtered through celite. The crude product was washed with sat. NH_4Cl, 0.1 M HCl, and brine (50 mL each) and evaporated under reduced pressure. The product was purified through flash column chromatography with cyclohexane/toluene 20:1 and isolated as a bright orange solid (582.5 mg, 89% yield). Melting point 274 ˚C. **^{1}H-NMR** ($CDCl_3$ 400 MHz): δ = (8.56 (s 4H), 8.33 (s 2H), 1.18 (s 24 H) 0.6 (s 36H) ppm. The data obtained matches data previously reported.[1b]

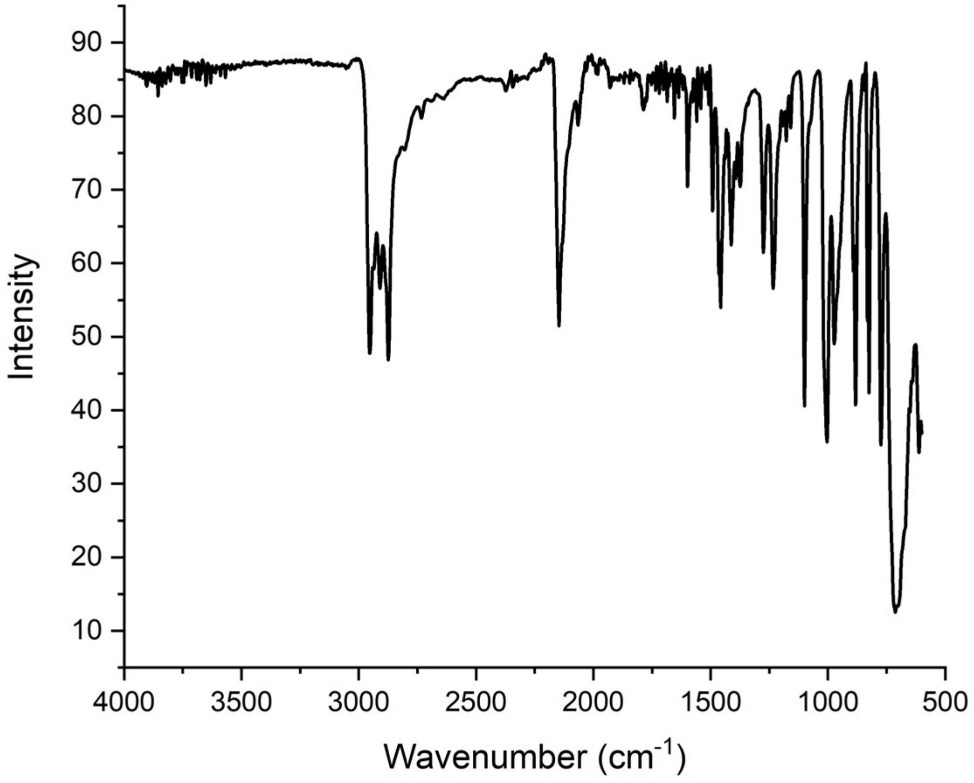

Figure S1: IR spectrum for 1,3,6,8-Tetra(triethylsilylethynyl) pyrene.

3.2 Synthesis of OSPC-Py

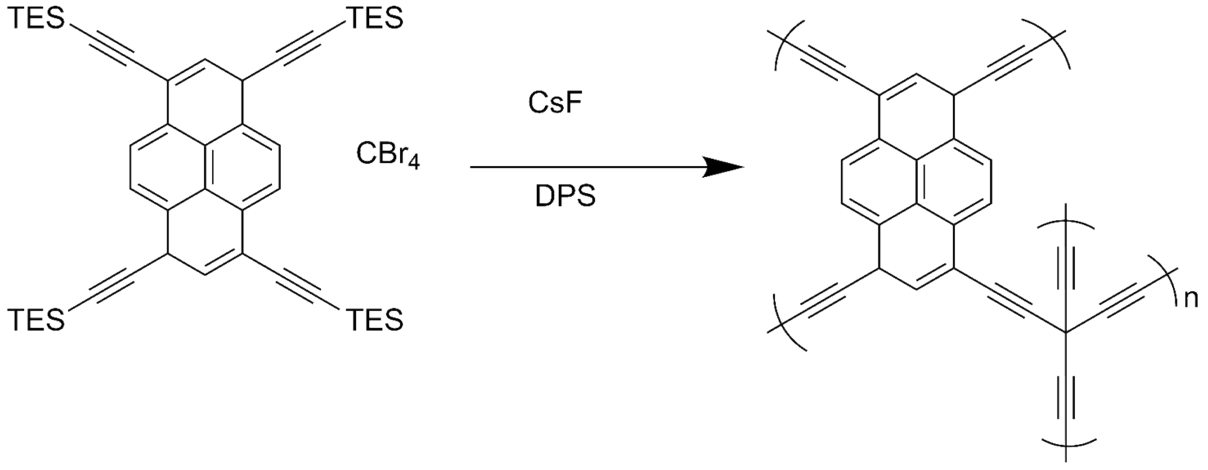

1,3,6,8-Tetra(triethylsilylethynyl) pyrene (0.295 g, 0.39 mmol), carbon tetrabromide (0.165 g, 0.5 mmol), CsF (0.375 g, 2.5 mmol) and DPS (2.5 g, solvent) were added to a sealed microwave vial under nitrogen. The mixture was heated at 200 ºC overnight. Then the reactor was cooled to 150 ºC and dichlorobenzene (5 mL) was added. The black precipitate was filtered out under reduced pressure and was suspended in NaOH solution overnight. The resultant solids underwent further purification using Soxhlet extraction with methanol. The products were dried under vacuum overnight to give a black powder of mass 168 mg. Assuming no residual end groups were present this would be a yield of 126%. Adjusting the yield using the EDX abundance data, as was done for OSPC-1 provides a lower bounded yield of 49%.[2]

Table S1: CHNS analysis of OSPC-Py. As the total is well below 100%, it is likely that the sample did not fully combust in the CHNS analyser. According to the TGA results shown in figure S1, over 60 % of the sample's mass remained at 900 °C. Another component of the remaining unassigned percentage may also be due to undetected bromine or silicon from unreacted end groups consistent with EDX analysis (Figure S6), which shows high relative abundance of both.

Sample	Mass (mg)	C (%)	H (%)	N (%)	S (%)	Total (%)
OSPC-Py	1.02	41.89	2.68	0.00	0.98	45.54
OSPC-Py	1.44	31.90	1.88	0.00	0.65	34.43

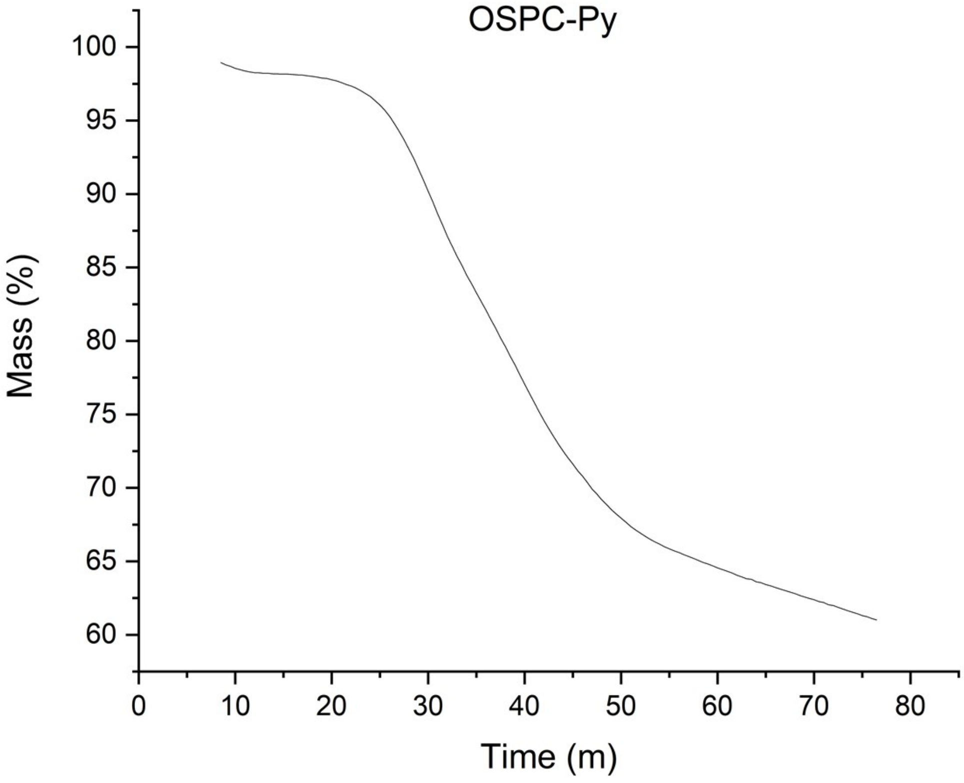

Figure S1: TGA results for OSPC-Py

3.2 Naming of OSPC-Py

Organically synthesised porous carbons (OSPCs) are a new class of materials that are a subset of conjugated microporous polymers (CMPs) that belong to the microporous organic polymer family of materials.[2]

We define an OSPC family member as their core polymeric repeat structure containing only carbon and that it is synthesised using an organic synthetic approach utilising monomeric units. This distinguishes it from other polymer materials, including CMPs and porous aromatic frameworks (PAFs) that contain hydrogen and other elements within their polymer repeat unit, and from other porous carbon materials, including hard carbons or amorphous carbons, that are not synthesised using an organic approach.

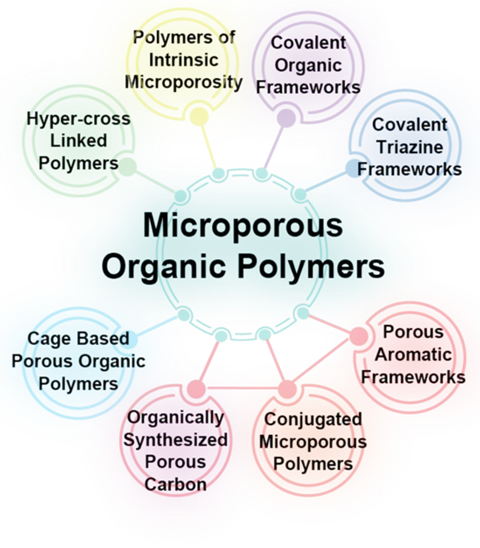

Panel S1: How OSPCs fit in to the family of microporous organic polymer materials. Reproduced from reference 2. [2]

In general, CMP materials are synthesised using the Sonogashira Hagihara carbon-carbon coupling approach, while PAFs are synthesised using the Yamamoto carbon-carbon coupling approach. Although other approaches are used, including Suzuki, Schiff-base, or cyclotrimerization approaches, these tend to produce materials that fit better into other categories such as hyper cross linked polymers. Whereas OSPC materials are synthesised using either a CsF catalyst, as we have done here, or via Eglington homo coupling, as undertaken in the first reported discovery of OSPC-1.[3]

Here we have incorporated pyrene units within the OSPC polymer structure demonstrating the flexibility of the synthetic approach. Incorporating this functionality means that by definition this material fits into the CMP family similarly to other pyrene based CMPs including YPy, YDBPy reported by 2011 by Jiang et al,[4] and the extension to this set incorporating phenyl linkers as *o*-phenyl-Py-CMP, *p*-phenyl-Py-CMP and *m*-phenyl-Py-CMP.[5]

There is no set rule for naming of CMP materials, with names being set by those that discover them to best suit their requirements. For OSPC materials, to-date these have been numbered to reflect the number of alkyne groups that link the tetrahedral sp3 carbons starting with OSPC-0, which has one linking alkyne unit and up to OSPC-3 that has four linking alkyne units.

As such, we name this material OSPC-Py to reflect the OSPC synthetic approach used, incorporate the pyrene functionality, distinguish it from OSPC materials (these are numbered) and from other pyrene containing CMP materials.

4. Computational generation of OSPC-Py cluster models

Ambuild is an in-house, GPU-based software specifically designed to model amorphous porous polymers such as CMPs, covalent triazine frameworks, porous aromatic frameworks and hypercrosslinked polymers.[6-10] It is written in Python and able to integrate with HOOMD-blue,[11, 12] used as the geometry optimisation and molecular dynamics (MD) engine throughout. As in our previous studies employing Ambuild, we utilised the polymer consistent (PCFF) forcefield to describe bonding and non-bonding interactions in the Ambuild models as this was deemed the most appropriate for our systems.[13] For further details on the Ambuild code, please see our previous publications (Reference set 3), where it is described in-depth, with full details and a validation of the approach, or its Github site (https://github.com/linucks/ambuild/wiki).

For the clusters, a tetrabromomethane (TBM) building block with all four end groups available for bonding is seeded into the centre of a cubic cell of size (50 Å, 50 Å, 50 Å) and the remaining cell volume filled with DPS solvent molecules to closely mimic the synthetic protocol. Then, a geometry optimisation is performed with a van der Waals cutoff of 10 Å and an integration timestep of 0.01 for 1000 cycles, followed by an *NVT* MD (constant number of molecules, cell volume and temperature) with a van der Waals cutoff of 10 Å, an integration timestep of 0.001 and a temperature factor of 55 for 100,000 cycles. The DPS is removed from the cell and a growBlocks step is performed to add a tetraethynylpyrene (TEP) building block to the original block *via* any of the available end groups, replacing a bromine cap atom.

The DPS is added back to the cell and a zipBlocks step is performed increasing the defined bond length and angle margins to 5 Å and 70° respectively, and determining whether the existing blocks in the system are close enough together to form additional bonds at the specified margins, and if so, these bonds are formed. Only bonds between a TEP and TBM are allowed to follow the synthetic chemistry. Another geometry optimisation and MD step is performed if the *zipBlocks* step was successful. This process is repeated, randomly growing a TEP or TBM onto available and allowed sites, until a 20-unit cluster is formed. This procedure was repeated 100 times, generating 100 independent clusters.

For the computational analysis, any unreacted bromine atoms present in the chains and clusters were replaced with hydrogen atoms to more realistically represent the small (approximate 4 wt.%) proportion of bromine atoms remaining after experimental synthesis.

CMPs are amorphous kinetic products and therefore not minimum-energy thermodynamic structures. Calculations on large chemical systems require careful consideration due to potentially significant computational expense. We have chosen computationally tractable characterisation techniques that provide information with a reasonable balance of accuracy and computational expense. The GFN2-xTB[14] semi-empirical tight binding approach, presented by Grimme and co-workers as an efficient electronic structure-based geometry optimiser for large chemical systems provides structures of an accuracy approaching those from conventional density-functional theory (DFT) calculations, was used within the XTB[15] program to optimise the geometry of the generated chains and clusters. This is considered a "relaxation" of the structure to obtain more sensible bond lengths, conjugation and intermolecular interactions than those provided by the basic rigid-body forcefield approach without imposing the inherent restrictions present in a molecular mechanics approach. Each of the relaxed structures were found to be minima on their respective potential energy surfaces. The harmonic vibrational normal modes are also calculated using this same model chemistry. We recently justified the applicability of the XTB-derived geometries for subsequent ^{13}C NMR and UV–vis spectroscopic analysis by considering the impact on several test systems and found that the difference between ^{13}C NMR and UV–vis data calculated from GFN2-xTB and B3LYP/cc-pVDZ geometries is minimal compared to using Ambuild structures.[16]

Single point energy calculations of the XTB-derived structures were undertaken using Gaussian 09 and the CAM-B3LYP/def2SVP model chemistry.[17-19] From this, molecular orbitals were generated.

5. Solid state NMR of OSPC-Py

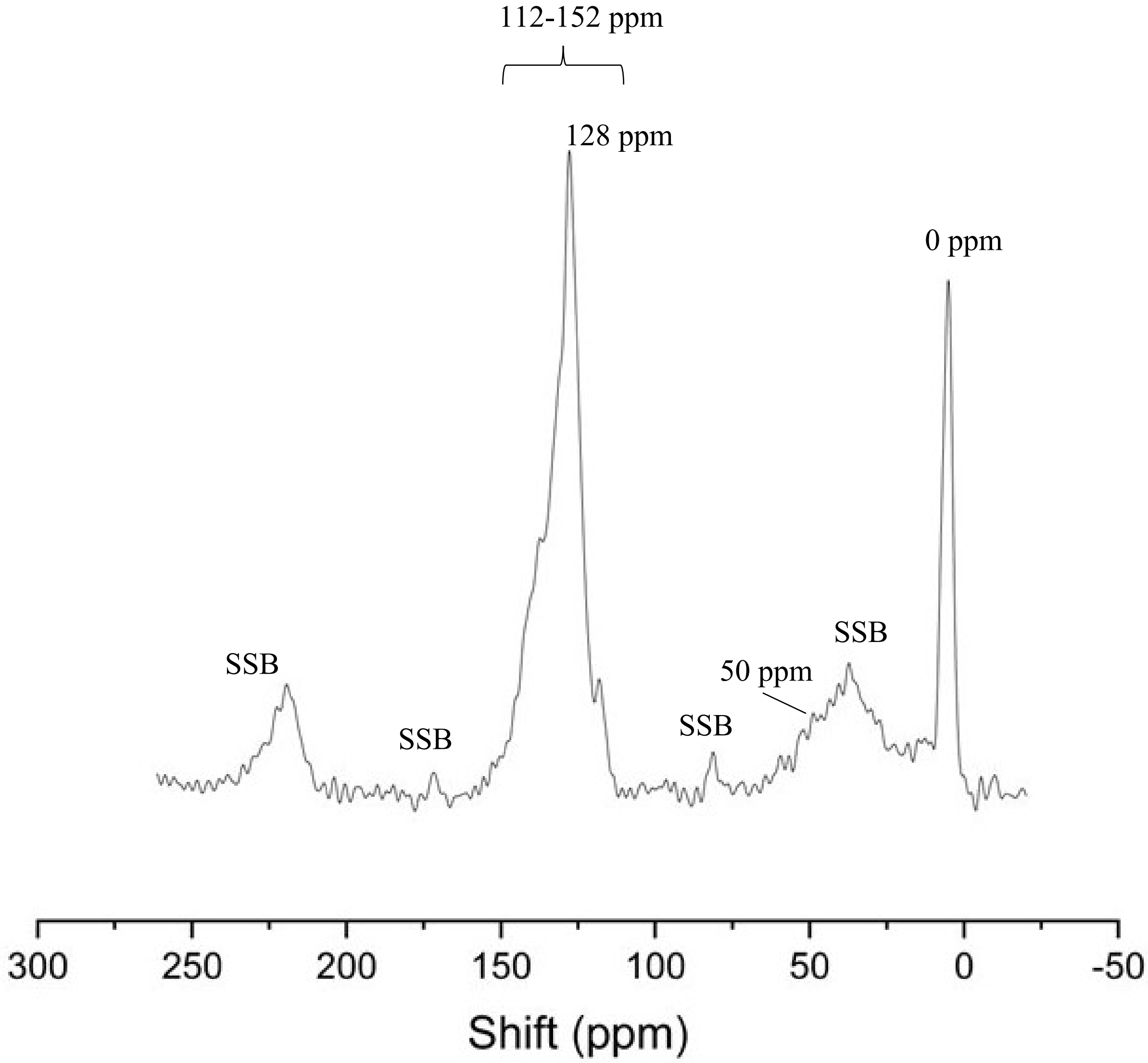

Figure S2: Solid state NMR spectra of OSPC-Py obtained using a MAS frequency of 16 kHz and the D1 was 3s. Pertinent peaks are labelled. Spinning side bands are labelled SSB.

6. UV/vis

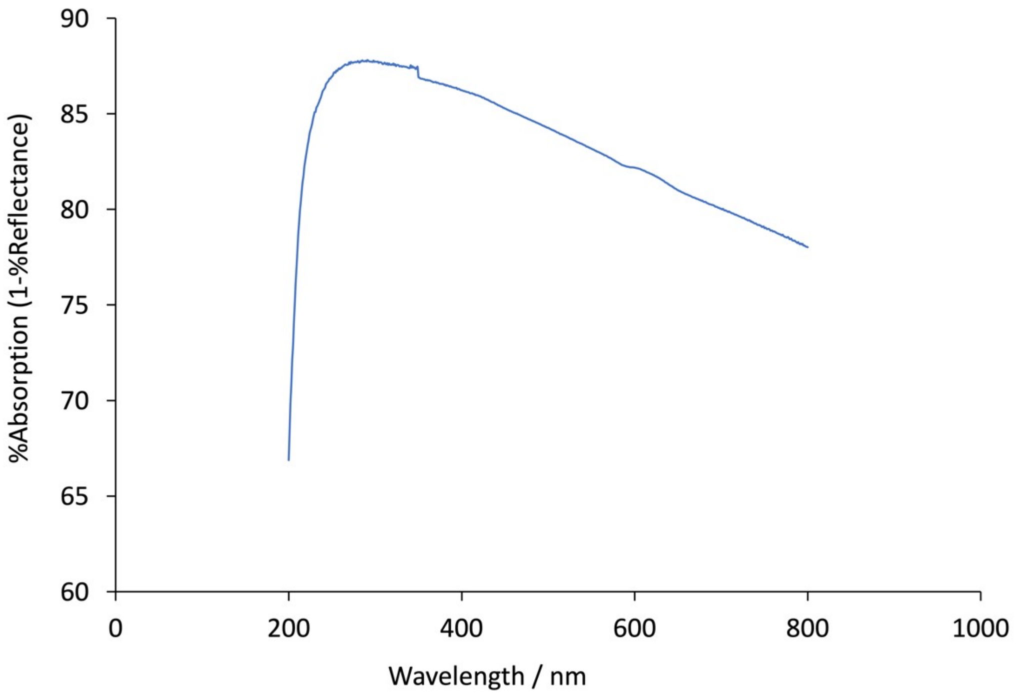

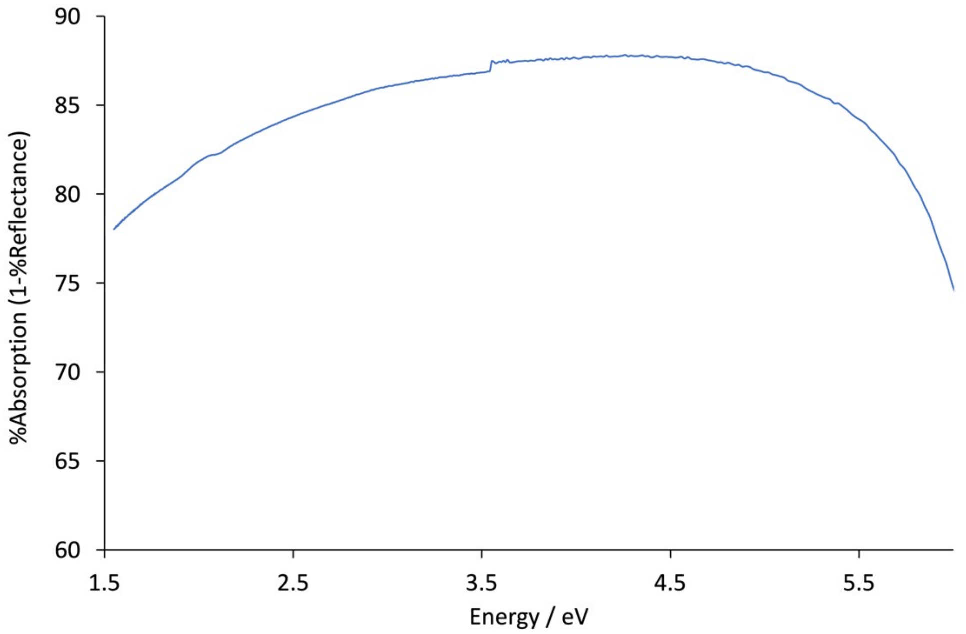

Figure S3: UV/vis spectra of OSPC-Py.

7. PSD analysis

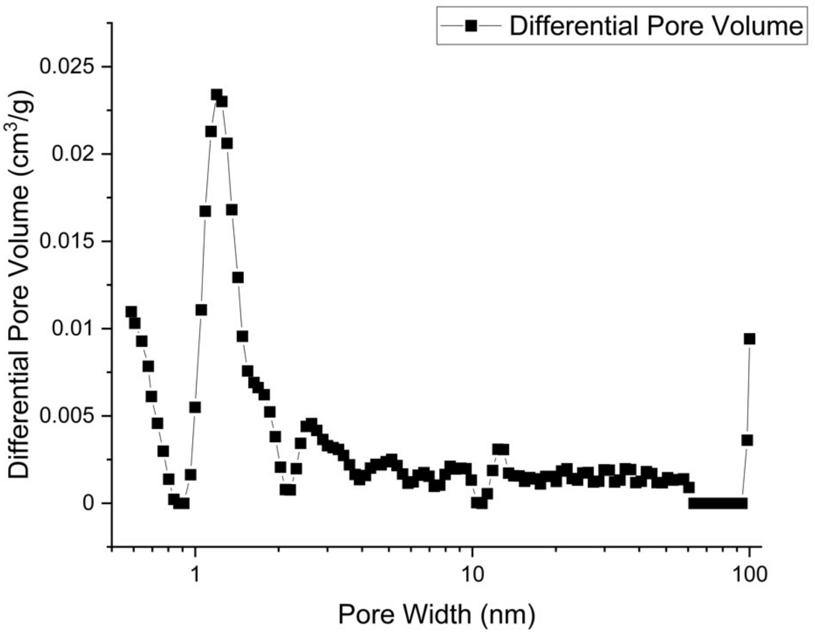

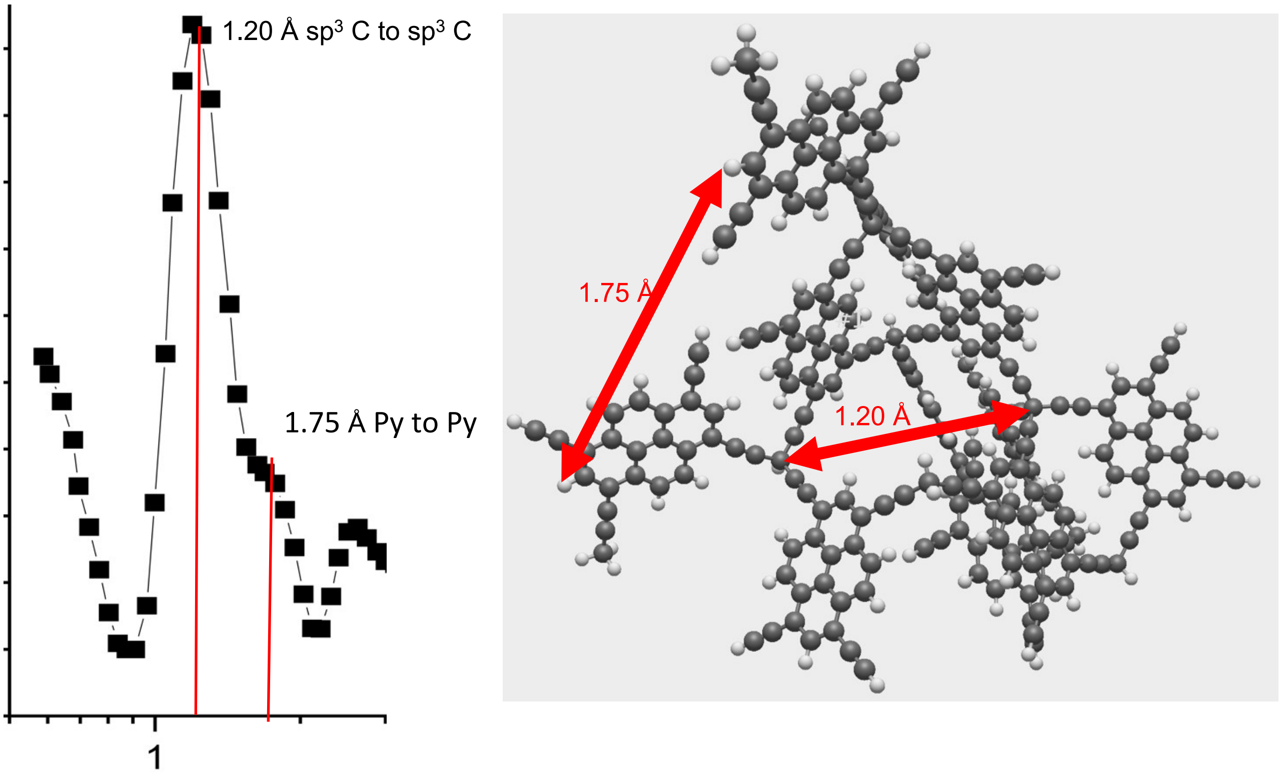

Figure S4: Pore size distribution analysis.

6. SEM

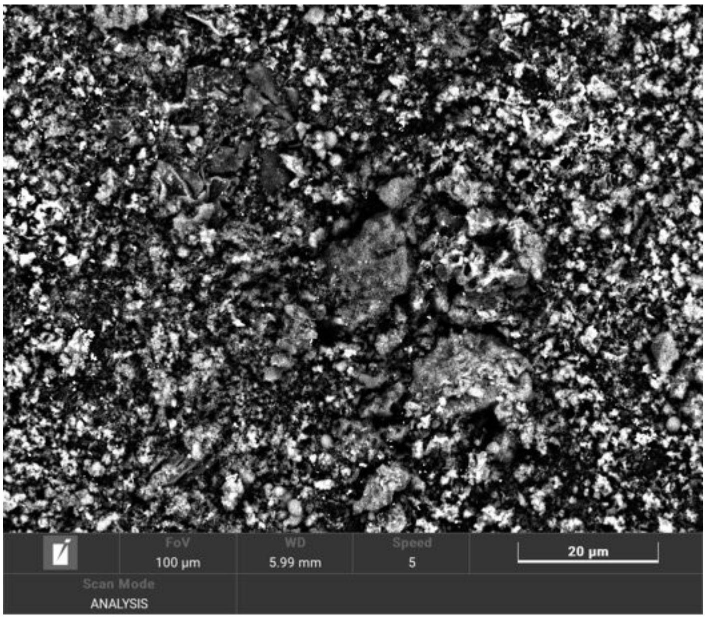

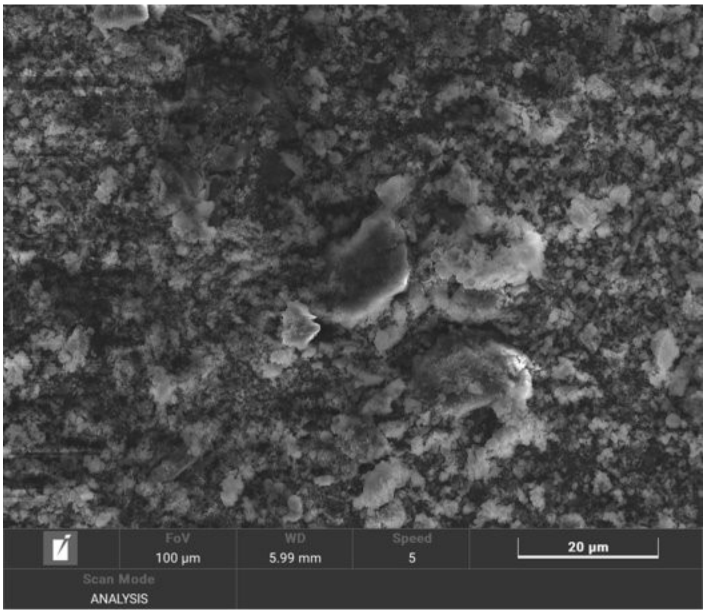

Figure S5: High resolution SEM with (top) and without (bottom) back scattering.

7. EDX

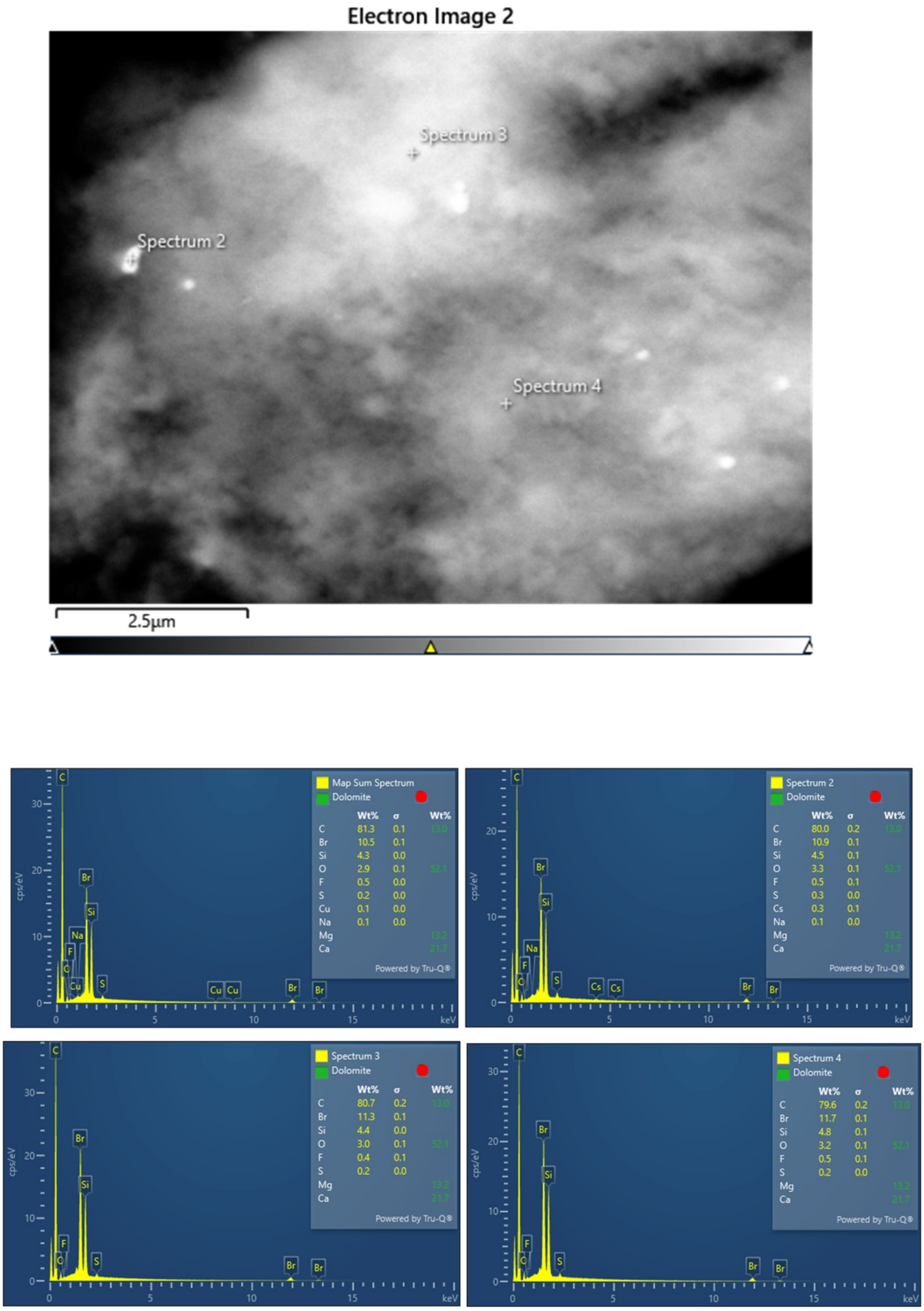

Figure S6: EDX data for the highlighted region. Site 3 is representative of the diffuse morphology whereas Site 4 is representative of the denser morphology. Site 2 was investigated as its prominence on the backscattered image gave the

appearance of an impurity, but it does not appear to have a different composition to the bulk structure. It may therefore be a particularly prominent example of the diffuse morphology.

8. Cluster Models

Table SI.1 The 100 unique 20-unit cluster models, their number of MCRs, the number of TEP units, and the number of TBM units

Model	Number of MCRs	Number of TBM	Number of TEP
**61**	0	14	6
**67**	1	7	13
**82**	1	8	12
**41**	1	9	11
**53**	1	9	11
**62**	1	9	11
**22**	1	10	10
**98**	1	10	10
**47**	1	11	9
**9**	1	12	8
**68**	1	12	8
**17**	2	7	13
**18**	2	7	13
**26**	2	7	13
**93**	2	7	13
**6**	2	8	12
**40**	2	8	12
**78**	2	8	12
**79**	2	8	12
**19**	2	9	11
**71**	2	9	11
**73**	2	9	11
**75**	2	9	11
**7**	2	10	10
**11**	2	10	10
**50**	2	10	10
**54**	2	10	10
**60**	2	10	10
**69**	2	10	10
**56**	2	11	9

**95**	2	11	9
**100**	2	11	9
**37**	2	12	8
**10**	2	13	7
**36**	2	13	7
**43**	2	13	7
**30**	2	14	6
**39**	3	7	13
**59**	3	7	13
**89**	3	7	13
**23**	3	8	12
**24**	3	8	12
**25**	3	8	12
**87**	3	8	12
**16**	3	9	11
**28**	3	9	11
**42**	3	9	11
**45**	3	9	11
**66**	3	9	11
**84**	3	9	11
**27**	3	10	10
**31**	3	10	10
**51**	3	10	10
**58**	3	10	10
**85**	3	10	10
**1**	3	11	9
**8**	3	11	9
**14**	3	11	9
**48**	3	11	9
**77**	3	12	8
**81**	3	12	8
**44**	3	13	7
**49**	3	13	7
**76**	3	13	7
**88**	3	13	7
**2**	4	8	12
**13**	4	8	12
**20**	4	8	12
**34**	4	8	12
**90**	4	8	12
**5**	4	9	11
**32**	4	9	11

**63**	4	9	11
**70**	4	9	11
**92**	4	9	11
**94**	4	9	11
**99**	4	9	11
**12**	4	10	10
**29**	4	10	10
**80**	4	10	10
**52**	4	11	9
**55**	4	11	9
**72**	4	11	9
**74**	4	11	9
**83**	4	11	9
**33**	4	12	8
**38**	4	12	8
**65**	4	14	6
**15**	5	8	12
**57**	5	9	11
**96**	5	9	11
**3**	5	10	10
**4**	5	10	10
**35**	5	10	10
**64**	5	10	10
**46**	5	11	9
**91**	5	11	9
**86**	6	8	12
**21**	6	12	8
**97**	7	10	10

Table SI.2 The four structural types identified.

**Structure ID**	**Number**
Branched	1
Single	10
Webbed	81
Disjoint	8

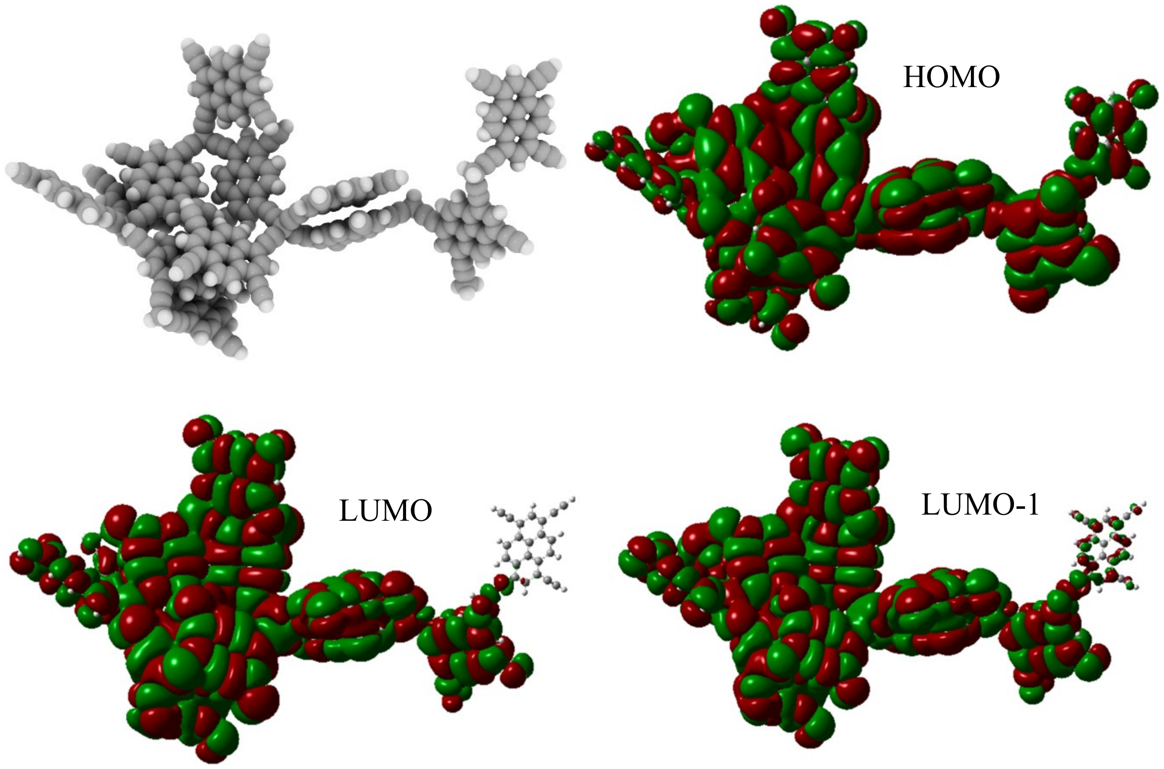

Figure S7: Model 22, an example of a model from the **Single** structural group visualised using iRASPA[20] and showing the HOMO, LUMO, and LUMO-1 molecular orbitals.

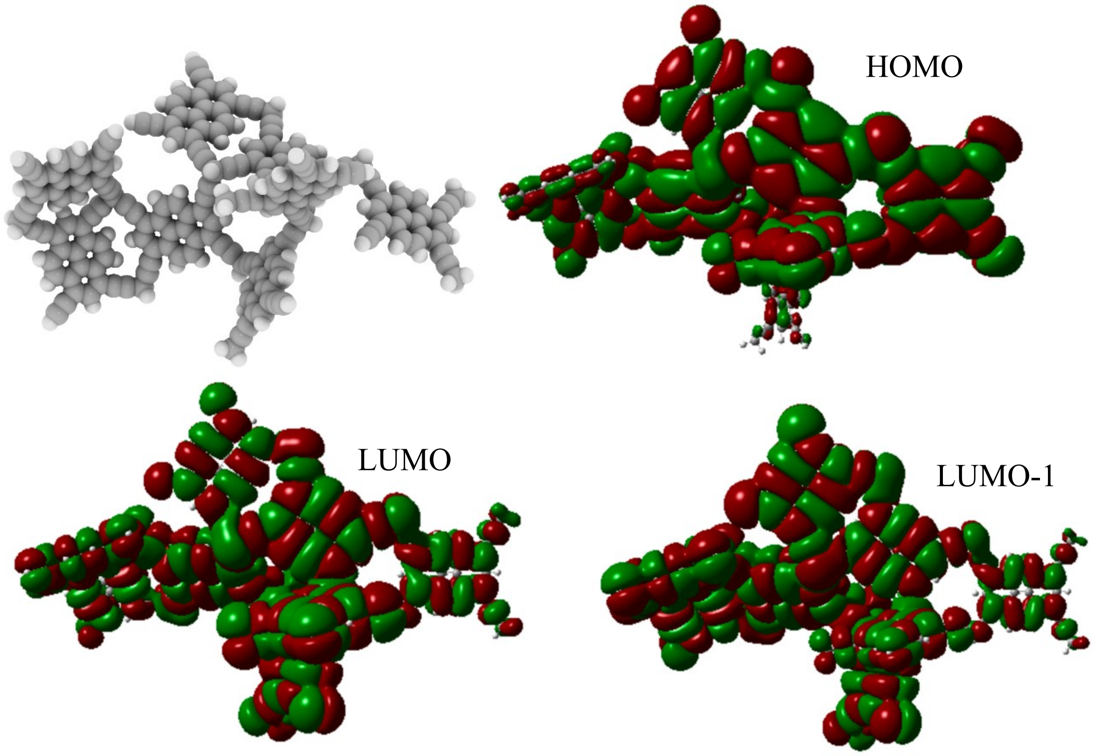

Figure S8: Model 2, an example of a model from the **Webbed** structural group visualised using iRASPA[20] and showing the HOMO, LUMO, and LUMO-1 molecular orbitals.

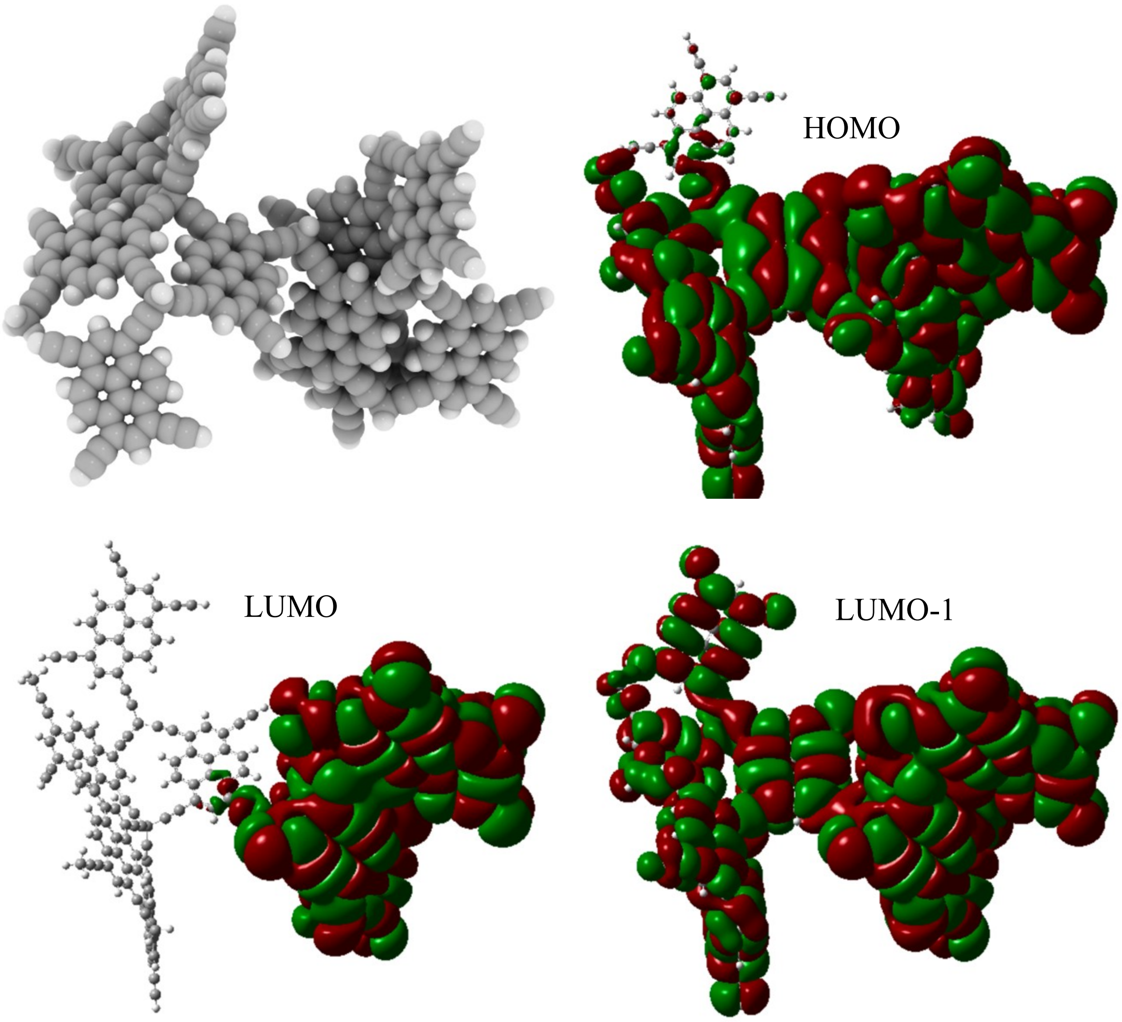

Figure S9: Model 31, an example of a model from the **Disjoint** structural group visualised using iRASPA[20] and showing the HOMO, LUMO, and LUMO-1 molecular orbitals.

References

(1a) Stegbauer, L.; Zech, S.; Savasci, G.; Banerjee, T.; Podjaski, F.; Schwinghammer, K.; Ochsenfeld, C.; Lotsch, B. V. Tailor-Made Photoconductive Pyrene-Based Covalent Organic Frameworks for Visible-Light Driven Hydrogen Generation. *Advanced Energy Materials* **2018**, *8* (24), 1703278. DOI: https://doi.org/10.1002/aenm.201703278 (acccessed 2025/11/10).

(1b) J.Isokuortti, C. J.O'Dea, S. R.Allen, S.Vasylevskyi, Z. A.Page, S. T.Roberts, Putting the "P" Back in Delayed Fluorescence – Silylethynyl Substitution Generates Efficient Pyrene Annihilators for Red-to-Blue Photon Upconversion. Adv. Optical Mater.2025, 13, 2500388. **https://doi.org/10.1002/adom.202500388**

(2) Rowling, A.; Doulcet, J.; Dawson, R.; Tapia-Ruiz, N.; Trewin, A. Facile Synthesis of Organically Synthesized Porous Carbon Using a Commercially Available Route with Exceptional Electrochemical Performance. *ACS Applied Materials & Interfaces* **2024**, *16* (36), 47631-47638. DOI: 10.1021/acsami.4c09710.
(3) Zhao, Z.; Das, S.; Xing, G.; Fayon, P.; Heasman, P.; Jay, M.; Bailey, S.; Lambert, C.; Yamada, H.; Wakihara, T.; et al. A 3D Organically Synthesized Porous Carbon Material for Lithium-Ion Batteries. *Angewandte Chemie International Edition* **2018**, *57* (37), 11952-11956. DOI: 10.1002/anie.201805924.
(4) Jiang, J.-X.; Trewin, A.; Adams, D. J.; Cooper, A. I. Band gap engineering in fluorescent conjugated microporous polymers. *Chem. Sci.* **2011**, *2* (9), 1777-1781, 10.1039/C1SC00329A. DOI: 10.1039/C1SC00329A.
(5) Mollart, C.; Heasman, P.; Sherrett, E.; Fletcher, P. A. T. J.; Fayon, P.; Thomas, J. M. H.; Franckevičius, V.; Peach, M. J. G.; Trewin, A. A New Combined Computational and Experimental Approach to Characterize Photoactive Conjugated 3D Polymers. *Small* **2025**, *21* (9), 2407187. DOI: https://doi.org/10.1002/smll.202407187.
(6) Fayon, P.; Thomas, J. M. H.; Trewin, A. Structure and Properties of a Nanoporous Supercapacitor. *J. Phys. Chem. C* **2016**, *120* (45), 25880-25891. DOI: 10.1021/acs.jpcc.6b08712.
(7) Liu, A.; Mollart, C.; Trewin, A.; Fan, X.; Lau, C. H. Photo-Modulating CO2 Uptake of Hypercross-linked Polymers Upcycled from Polystyrene Waste. *ChemSusChem* **2023**, *16* (10), e202300019. DOI: https://doi.org/10.1002/cssc.202300019.
(8) Mollart, C.; Holcroft, S.; Peach, M. J. G.; Rowling, A.; Trewin, A. Artificial synthesis of covalent triazine frameworks for local structure and property determination. *Phys. Chem. Chem. Phys.* **2022**, *24* (34), 20025-20029, 10.1039/D2CP02430F. DOI: 10.1039/D2CP02430F.
(9) Thomas, J. M. H.; Mollart, C.; Turner, L.; Heasman, P.; Fayon, P.; Trewin, A. Artificial Synthesis of Conjugated Microporous Polymers via Sonogashira–Hagihara Coupling. *J. Phys. Chem. B* **2020**, *124* (33), 7318-7326. DOI: 10.1021/acs.jpcb.0c04850.
(10) Thomas, J. M. H.; Trewin, A. Amorphous PAF-1: Guiding the Rational Design of Ultraporous Materials. *J. Phys. Chem. C* **2014**, *118* (34), 19712-19722. DOI: 10.1021/jp502336a.
(11) Anderson, J. A.; Lorenz, C. D.; Travesset, A. General purpose molecular dynamics simulations fully implemented on graphics processing units. *J. Comput. Phys.* **2008**, *227* (10), 5342-5359. DOI: https://doi.org/10.1016/j.jcp.2008.01.047.
(12) Glaser, J.; Nguyen, T. D.; Anderson, J. A.; Lui, P.; Spiga, F.; Millan, J. A.; Morse, D. C.; Glotzer, S. C. Strong scaling of general-purpose molecular dynamics simulations on GPUs. *Comput. Phys. Commun.* **2015**, *192*, 97-107. DOI: https://doi.org/10.1016/j.cpc.2015.02.028.
(13) Sun, H. Ab initio calculations and force field development for computer simulation of polysilanes. *Macromolecules* **1995**, *28* (3), 701-712. DOI: 10.1021/ma00107a006 (acccessed 2012/08/13).

(14) Bannwarth, C.; Ehlert, S.; Grimme, S. GFN2-xTB—An Accurate and Broadly Parametrized Self-Consistent Tight-Binding Quantum Chemical Method with Multipole Electrostatics and Density-Dependent Dispersion Contributions. *J. Chem. Theory Comput.* **2019**, *15*, 1652–1671.
(15) Bannwarth, C.; Caldeweyher, E.; Ehlert, S.; Hansen, A.; Pracht, P.; Seibert, J.; Spicher, S.; Grimme, S. *WIREs Comput. Mol. Sci.* **2020**, *11*, e01493.
(16) Mollart, C.; Heasman, P.; Sherrett, E.; Fletcher, P. A. T. J.; Fayon, P.; Thomas, J. M. H.; Franckevičius, V.; Peach, M. J. G.; Trewin, A. A new combined computational and experimental approach to characterise photoactive conjugated 3D polymers. *Small* **2025**, *Early View*, 2407187.
(17) Yanai, T.; Tew, D. P.; Handy, N. C. A new hybrid exchange–correlation functional using the Coulomb-attenuating method (CAM-B3LYP). *Chemical Physics Letters* **2004**, *393* (1), 51-57. DOI: https://doi.org/10.1016/j.cplett.2004.06.011.
(18) Weigend, F.; Ahlrichs, R. Balanced basis sets of split valence, triple zeta valence and quadruple zeta valence quality for H to Rn: Design and assessment of accuracy. *Physical Chemistry Chemical Physics* **2005**, *7* (18), 3297-3305, 10.1039/B508541A. DOI: 10.1039/b508541a.
(19) Frisch, M. J.; Trucks, G. W.; Schlegel, H. B.; Scuseria, G. E.; Robb, M. A.; Cheeseman, J. R.; Scalmani, G.; Barone, V.; Mennucci, B.; Petersson, G. A.; et al. Gaussian 09 Revision E.01. 2015 Gaussian 09 Revision E.01.
(20) Dubbeldam, D.; Calero, S.; Vlugt, T. J. H. iRASPA: GPU-accelerated visualization software for materials scientists. *Molecular Simulation* **2018**, *44* (8), 653-676. DOI: 10.1080/08927022.2018.1426855.
